# Spontaneous Subdural Hematoma and Behavioral Changes Due to a Dural Arteriovenous Fistula. A Case Report and Literature Review

**DOI:** 10.3390/bs9060063

**Published:** 2019-06-14

**Authors:** Zenaida Milagros Hernández-Díaz, Juan Carlos Llíbre-Guerra, Marianela Arteche-Prior, Tania de la Paz-Bermúdez, Angel Francisco Águila-Ruiz, Luisa María Figueroa-García, María de los Ángeles Robinson-Agramonte

**Affiliations:** 1 Department of Neuroimagenology, International Center for Neurological Restoration, 25th Ave, Playa 15805, Havana 11300, Cuba; taniapaz@neuro.ciren.cu (T.d.l.P.-B.); angel@neuro.ciren.cu (A.F.Á.-R.); robin@neuro.ciren.cu (M.d.l.Á.R.-A.); 2 Department of Endovascular Therapy and Stroke Unit, Institute of Neurology and Neurosurgery, Calle 29 y Esquina D, Havana 10400, Cuba; juancarlos.llibre@gmail.com (J.C.L.-G.); artecheprior@infomed.sld.cu (M.A.-P.); luisamar@infomed.sld.cu (L.M.F.-G.)

**Keywords:** behavioral changes, dural arteriovenous fistula, intracerebral hematoma, subdural hematoma, rare diseases

## Abstract

Dural arteriovenous fistulas (DAVFs) represent 10–15% of intracranial arteriovenous malformations. Of these, only 12–29% cause intracranial hemorrhage. The presentation of DAVF as a subdural hematoma (SDH) and intraparenchymal hemorrhage (IPH) is infrequent; additionally, behavioral changes are not common among these patients. We report, for the first time in our country, the case of a 23-year-old man with no history of head injury, in which a brain computed tomography (CT) scan revealed SDH and IPH with behavioral disturbances. The angiotomography showed ecstatic venous vessels, indicating the presence of a DAVF, which was later confirmed by cerebral angiography. Endovascular therapy, which followed the clinical diagnosis, resulted in satisfactory evolution two years after treatment. A review of the literature concerning cases with DAVF and behavioral disturbances is presented. DAVF may lead to cognitive impairment, behavioral changes, and dementia as a result of diffuse white matter and thalamus modifications related to venous ischemia, and it should be considered as a reversible cause of vascular dementia.

## 1. Introduction

Dural arteriovenous fistulas (DAVFs) are defined as abnormal arteriovenous communications in which dural arteries that originate from the branches of the carotid or the vertebral arteries drain directly into a dural vein or the sinus [1,2,3,4]. DAVFs represent 10–15% of all intracranial malformations. In a DAVF, the shunt is located exclusively in the pachymeninx and the nest is often close to a dural sinus that may appear narrowed or obstructed in angiography. Venous drainage occurs through the dural sinus, and the dural or leptomeningeal ducts, distinguishing them from pial and parenchymal arteriovenous malformations [1,2].

DAVFs have multiple causes. Those of congenital origin are due to persistent embryological/fetal vessels, or to genetic alterations, such as phacomatoses and Galen’s vein malformation. Meanwhile, acquired DAVFs can be traumatic, the most common of which is the direct carotid-cavernous fistula, following cranial fractures, and involving the middle meningeal artery crossing the temporal scale, and mild cranioencephalic trauma. Other causes are secondary to surgery, direct or remote from craniotomy, tumor invasion of dural sinuses (e.g., meningiomas), pregnancy, infection, drugs (e.g., oral contraceptives), coagulopathies/hemoglobinopathies, and idiopathic [3]. The presentation of dural fistulae as spontaneous subdural intraparenchymatous hematomas is uncommon in general, especially the spontaneous hemorrhagic form. The patient documented in this study represents the first case reported in our country presenting with both subdural hematoma (SDH) and intraparenchymal hemorrhage (IPH) and behavioral changes.

## 2. Method

A literature search on MEDLINE was conducted for journal articles published from 1998 to 2019. The most common keywords employed were as follows: dural arteriovenous fistulas, endovascular treatment, behavioral disturbances, cognitive impairment, and dementia. All articles found through this database were reviewed, and those meeting the inclusion criteria were included in this study. The inclusion criteria were as follows: the availability of full texts, well-described cognitive, clinical, and neurological symptomatology, as well as imaging and treatment description. The investigation was in accordance to the 2008 revised version of the Declaration of Helsinki (1975) and the report was approved by Record 12/2018, given by the Ethics Committee of Institute of Neurology and Neurosurgery. A previous informed consent was necessary to be included in the investigation.

## 3. Clinical Case

A 23-year-old male with a history of migraine headaches for over 10 years was studied. The headaches responded favorably to usual analgesic treatment and worsened with physical exercise.

The patient looked for medical attention due to sudden and intense headaches in the occipital region with no evident cause, whose characteristics differed from the previous ones. An emergency computed tomography (CT) scan was performed, revealing the presence of a right occipital intracerebral hematoma that required immediate hospitalization and treatment for a month until resolution of the clot. One year after being symptom-free, he began to experience a mild to moderate oppressive headache in the right temporal region, which improved with analgesics for approximately 15 days. After that, the oppressive headache restarted with more intense hemicrania, accompanied by fatigue, short episodes of altered consciousness, sweating, multiple instances of projectile vomiting, and blurred vision of the left eye. General physical and neurological exams did not show any positive data, nor was there evidence of triggering events, such as traumatic brain injury, hematologic disorders, previous surgeries, or infections of the central nervous system (CNS).

Mood changes, including permanent irritability, aggressiveness, anxiety, diminished ability to think or concentrate, and temporo-spatial time disorientation, appeared during the follow-up period and a second CT-scan was performed.

### 3.1. Laboratory Studies

Laboratory tests included the following: hemoglobin 133 g/L; leucocytes 8.1 × 10^3^, platelets 373 u/L. A coagulogram indicated the following: suitable platelets, bleeding time 1.30 s, coagulation time 8 min, prothrombin time 15.2 s (control 13 s ± 3), and activated thromboplastin time 30 s; glycemia 4.83 mmol/L; glutamic oxaloacetic transaminase (GOT) 42.51 U/L; glutamic oxaloacetic transaminase (GPT) 42.29 U/L; cholesterol 4.24 mmol/L; triglycerides 0.90 mmol/L; creatinin 82.81 μmol/L; uric acid 280 μmol/L; total protein 72.15 g/L; albumin 41.30 g/L; negative human immunodeficiency virus (HIV) and non-reactive Venereal Disease Research Laboratory (VDRL).

### 3.2. Imaging Studies

**Figure 1:**
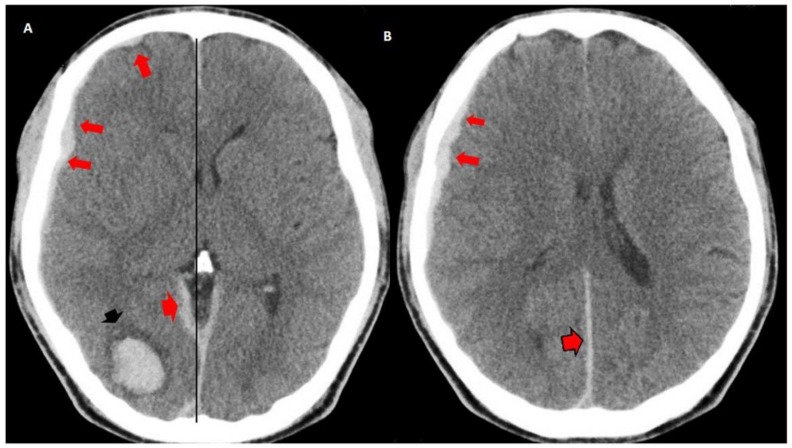
Simple brain computed tomography (CT): intraparenchymal hematoma in the right occipital region (black arrow head in (**A**)). Right frontal laminar subdural hematomas: (red arrows in (**A**,**B**), subdural hematoma at the tent of the cerebellum (red arrow head) and posterior interhemispheric subdural hematoma in contact with the sickle (red arrow head with black border in (**B**)). Mild mass effect on the ipsilateral ventricle, effacement of the subarachnoid space. Midline displaced 0.5 cm.

Urgent multislice tomography showed a laminar SDH in the right fronto-parietal region, a posterior interhemispheric fissure at the level of the tentorium, and ipsilateral occipital intracerebral hematoma (2.2 × 2.3 cm in axial section), with a volume of 7 mL. These lesions produced a slight mass effect on the neighboring structures, and the midline was displaced 0.5 cm (Figure 1).

**Figure 2:**
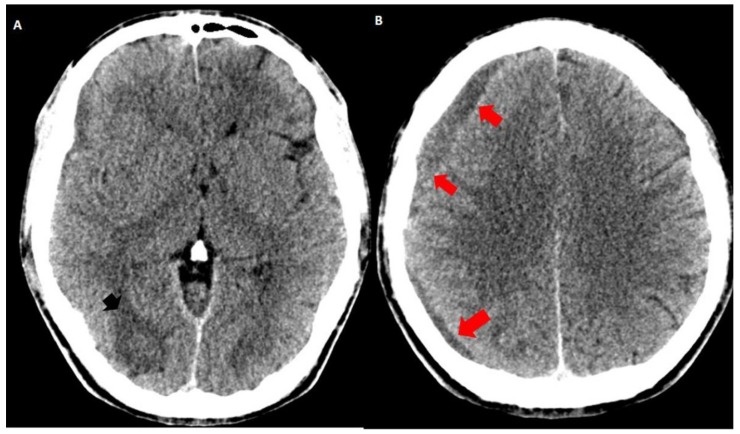
Simple brain CT one month later: the increased hypodensity of the intraparenchymal (black arrowhead in (**A**)) and subdural hematomas (red arrows in (**A**,**B**)) indicate hemoglobin degradation. The frontal subdural hematoma slightly increased its volume and extended to the right parietal region (red arrows).

**Figure 3:**
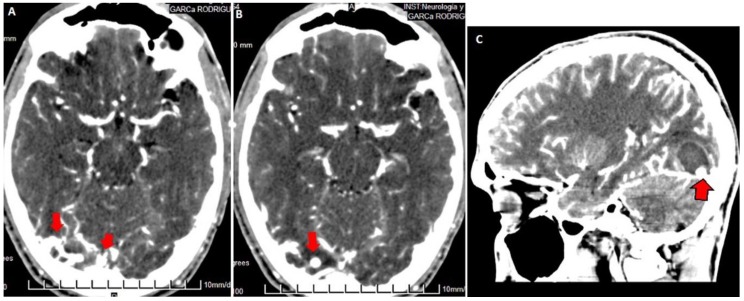
Contrasted brain CT: in axial section (**A**,**B**) dilated vessels are observed in the right occipital region (red arrows in (**A**)) with saccular venous ectasia (red arrows in (**B**,**C**)). Sagittal reconstruction (**C**) shows intraparenchymal hematoma in resolution with a ring-like enhancement on its periphery. The dilated saccular vein is in contact with the lower edge of the hematoma (red arrow with a black border).

**Figure 4:**
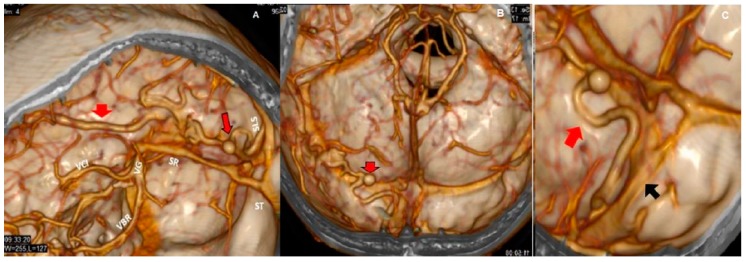
3D CT reconstructions of vessels and sinus (**A**–**C**). The saccular dilatation (red arrows with black borders in **A**,**B**) and drainage veins (red arrows in **A** and **C**) go toward the superior longitudinal sinus; the transverse sinuses are permeable. The upper longitudinal sinus (SLS, black arrow in **C**) at the level of the Herophilus prensa is fenestrated by arachnoid granulations or partial thrombosis that narrow it. The internal cerebral veins (VCI), basal veins of Rosenthal (VBR), vein of Galen (VG), rectum sinus (SR), and transverse sinuses (ST) all have a normal caliber.

**Figure 5:**
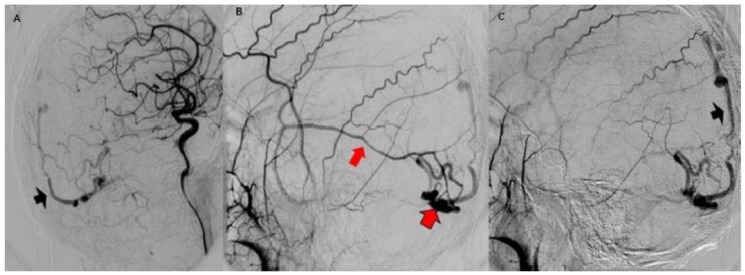
Digital subtraction angiography. (**A**) branch of the external carotid is seen as the afferent vessel (red arrow in (**B**)) to the malformative nest (red arrow with black border in (**B**)) with a thick vein of early cortical drainage (black arrowheads in (**A**,**C**)) that goes to the longitudinal superior sinus.

**Figure 6:**
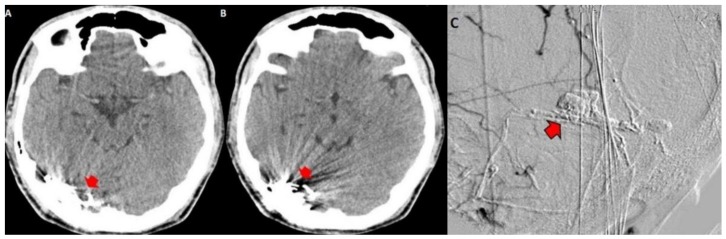
Contrasted CT showing embolized dural arteriovenous fistula with Onyx by endovascular procedure. This material produces artifacts with image distortion in the right occipital region (red arrow heads in (**A**,**B**)). The angiography after the therapeutic procedure of the fistula shows that it was successfully embolized, so it is not contrasted (red arrow with black outline in (**C**)).

Both hematomas were reabsorbed gradually (Figure 2), with satisfactory clinical evolution after a two-month follow-up period. Figure 2 shows the increased frontal subdural hematoma and its extension to parietal and frontal lobes. Angiography revealed ecstatic cortical veins, one of them with saccular dilatation adjacent to the IPH (Figure 3). For this reason, we decided to perform cerebral angiography, which confirmed the dural arteriovenous fistula. It connected the artery, a branch of the external carotid artery, with cortical veins in the superior sagittal sinus drainage (Figure 4). After endovascular therapy, the evolution of the patient was satisfactory (Figure 5 and Figure 6). Two years later, no bleeding had appeared, and the subsequent angiographic studies were negative.

## 4. Discussion and Literature Review

The first to describe an arteriovenous malformation were Rizzoli in 1881 and Sachs in 1931; the later demonstrating it through angiography [4]. In 1972, Houser distinguished between direct drainage in venous sinuses and drainage in subarachnoid pial veins, while in 1978 Djindjian and Merlan performed a general classification that correlated venous drainage patterns with symptomatology [4,5]. Dural fistulas are infrequent in the general population. A 27-year study in Minnesota reported an incidence of 0.15 per 100,000 people per year [6], while a Japanese study showed a detection rate of 0.29 per 100,000 people per year [7]. The number of cases in the different series reported is extremely limited due to its rarity [8].

Although dural arteriovenous fistulas are diagnosed more frequently between 40 and 50 years of age, they can occur at any age [9]. Our report refers to a 23-year-old adult man, in whom a diagnosis of AVF of possible congenital or idiopathic etiology was established, without any other cause to justify the mild headaches registered in his life history. This patient had a favorable evolution with the consumption of analgesics for approximately 12 years, after which he presented with intense hemicrania headache, accompanied by behavioral changes such as irritability, aggressiveness, diminished ability to think or concentrate, and disorientation in time and place.

Dural arteriovenous intracranial fistulas have different forms of clinical presentation: headache, pulsatile tinnitus, exophthalmos, non-hemorrhagic focal neurological deficit, epilepsy, or motor defect. The severity of symptoms depends on the venous drainage, and 12–29% bleed [10,11,12]. In a systematic review of the literature, Hou et al. reported 33 studies involving 42 patients, of whom 9 (21.4%, 9/42) patients presented with cognitive impairment [11]. Hemorrhages are one of the most serious clinical manifestations, appearing with loss of consciousness, motor defects, and can even cause death [10,12]. In a published meta-analysis, 20 studies involving 2513 patients were reported: 269 patients (11%) received symptomatic treatment with a follow-up period between 1 and 210 months; 9 of the patients (3.3%) had intracranial hemorrhages and 7 (2.6%) died [8]. The risk of intracranial hemorrhage associated with DAVF was between 1.8% and 3.7%. This study also showed that patients with intracranial bleeding had an increased risk of relapse (7.4%) as compared with those that had no previous hemorrhage (1.5%) [8]. This meta-analysis supports the relevance of evaluating patients with spontaneous intracranial bleeding of unknown cause, because they could be carriers of a DAVF and subsequent bleedings may threaten their lives.

Intracranial hemorrhage can be present in different compartments, generally in the subarachnoid intraparenchymatous or intraventricular space and less frequently in the subdural space [13,14]. Li C et al. [15] reported in a series of 236 patients that only 23.6% (56 patients) presented with intracranial hemorrhage at onset; 34 of these patients (60.7%) had intraparenchymal hemorrhage, 13 (23.2%) subarachnoid, 9 (16.1%) intraparenchymal and intraventricular hemorrhage, and only 2 (3.6%) subdural hematoma. The report from these authors endorses the infrequent debut of DAVF as a subdural hematoma [14,15,16,17], and further reinforces the importance of considering this condition, especially because the severity of the clinical picture. It is particularly important to consider the unusual presentation of subdural hematomas related to DAVF without having an identified traumatic cause.

Duffau et al. [18] reported four patients (three males and one female), all of whom were older than 55 years. Two of them presented with SDH at onset and the rest with IPH and SDH. Two of the cases evolved satisfactorily after receiving endovascular and surgical treatment, and two died. Kohyama et al. [17] reported the case of a 60-year-old patient who presented with headache. The subdural hematoma was evidenced by CT scan, with satisfactory evolution after endovascular and surgical treatment. Kitazono et al. [19] and Saito et al. [20] reported two male patients (aged 68 and 56 years). The first one presented headache at onset without any physical examination findings, but the other presented upheaval of consciousness leading eventually to coma. The CT scan identified SDH and occipital intraparenchymal hematoma in both cases; one of the patients was treated only with surgery and the other was treated with a combination of endovascular therapy and surgery, with a satisfactory outcome. Ogawa [14] and Kominato [21] reported two patients with subdural hematoma: one underwent surgical intervention, and had a favorable outcome, but the other patient, who was not treated, died.

Intracranial venous hypertension plays a significant role in the development of dural fistula, involving vessel radius and the draining vein, while clinical behavior depends mainly on the venous drainage patterns and risk of intracranial hypertension and hemorrhage. Cortical venous drainage predisposes to a more aggressive clinical course [5,22]. The patient in this report presented a DAVF type IV according to Cognard´s classification and type III according to Borden´s classification, both of which are considered to be responsible for the intracranial hemorrhage. The saccular dilatation of the venous ectasia, located above the tentorium and adjacent to the intraparenchymal occipital hematoma, was the point where the cortical vein wall was fissured. The increased size of the hematoma produced compression over the frontal and parietal lobes, together with endocraneal hypertension, which could be related to most of the behavioral changes experienced by the patient.

Cognitive impairment, affecting the domains of memory, calculation, orientation, visuospatial function, and language, is also present in patients with DAVF [23,24]. Several reports have demonstrated the presence of cognitive disturbances in patients with DAVF [2,23,24,25,26,27,28,29,30]. The most prevalent symptoms reported were disorientation in time and place, inability to walk, amnesia and alexia, deterioration of short-term memory, forgetfulness of peoples’ names, and difficulty with reading and the performance of activities [23,29]. The most frequent symptoms found in this case report were irritability, disorientation in time and place, and anxiety, which have also been reported by other authors in DAVF patients [25,28] (Table 1).

Most of the reports of patients with DAVF and cognitive impairment showed abnormal Magnetic Resonance Imaging (MRI) signals associated with mild swelling, focal edema, and white matter hyperintensities [2,23,27,29], while single photon emission computed tomography (SPECT) studies have demonstrated a decreased cerebral blood flow in different areas of the brain [23,29]. However, in the patient described in this report, the increased hematoma size and endocraneal hypertension were the surrogate causes of his behavioral changes. Almost all subjects described in case reports were treated with surgery and endovascular embolization, and cognitive impairment was improved, with resolution of most of the cognitive symptoms. In addition, the return of patients to work or daily activities without impairment and the complete improvement of the executive functions have been frequently reported [25,30]. In fact, cognitive impairment from DAVFs, termed variably ‘encephalopathy’ or ‘altered mental status’ in the literature, has been described as a consequence of venous hypertension or ischemia and may be reversible in most cases with early treatment [28] (Table 1).

On the other hand, dementia in patients with DAVF is frequent and has been described in various case reports and case series [31,32,33,34,35,36,37]. The principal factor for dementia in DAVF cases is the cortical venous drainage, which produces venous congestion and hypertension, leading to congestive encephalopathy. This venous hypertension reduces the normal pressure gradient between arteries and veins, which occurs most often in the deep venous system; as a result, white matter is the most vulnerable, leading to venous ischemia and the development of subcortical dementia [33] (Table 1).

Diffuse deep and subcortical white matter lesions [27,33,34,35,37] and SPECT hypoperfusion [32] are the imagenological features most commonly described. In a series of 40 DAVF patients, the development of dementia because of venous hypertensive encephalopathy was evaluated. Five of the patients showed dementia, and in all cases, MRI showed abnormalities in parenchymal cerebral regions, which were remote from the site of the DAVF, with areas of edema or venous ischemia [26] (Table 1).

However, a case reported by Geraldes et al. showed progressive cognitive impairment, ataxia, and myoclonus in a 64-year-old man with a DAVF. The clinical symptoms were explained by venous hypertension in the deep venous system, producing bilateral basal ganglia/thalamic dysfunction, without involvement of deep white matter [38] (Table 1).

In addition, DAVF cases with infrequent neurological manifestations, such as gait ataxia, parkinsonism, and myoclonus, have also been reported [32,35,39,40,41,42]. In most of these reports, SPECT hypoperfusion in basal ganglia and diffuse deep white matter are the most common findings [32,40]. Gradual impairment in cerebral circulation due to venous hypertensive encephalopathy could be involved in slowly progressive dementia with leukoencephalopathy in patients with DAVF [43] (Table 1).

The occurrence of parkinsonism together with progressive cognitive dysfunction caused by DAVF has been rarely reported. However, Luo et al. reported two DAVF cases with parkinsonism and progressive cognitive dysfunction [44]. The authors suggested that the underlying pathophysiology could be due to venous hypertension caused by DAVF leading to basal ganglia and cortical dysfunction [44]. Matsuda et al. reported three DAVF patients with progressive dementia and parkinsonism, suggesting that these symptoms could be caused by diffuse cerebral parenchymal disturbance and impaired cerebral circulation due to severe venous hypertension [34]. Abnormal gait and balance resulting in falls was reported in a DAVF patient with hyperintense signals in the brainstem [45]. Occasionally, patients with DAVF could present focal neurologic deficits, a dementia-like syndrome, hemorrhage, or ischemic infarction [46] (Table 1).

In addition, some studies of DAFV reported behavioral problems and decline in the activities of daily life [24,47], as well as attention deficit and emotional lability [48]. In these cases, after endovascular treatment the patients were able to take care of themselves completely [47].

## 5. Conclusions

This case report shows the need to rule out DAVF in patients with spontaneous acute subdural and intra-parenchymatous hematomas, with behavioral disorders and/or cognitive impairments. In addition, early endovascular treatment should be applied to DAVF, because DAVF may lead to dementia with diffuse white matter changes related to venous ischemia and should be considered as a reversible cause of vascular dementia.

## Figures and Tables

**Table 1:** Series of published cases that report patients with DAVF, behavioral and cognitive impairment.

Source	Total Patients	Cognitive Impairment	Dementia	Parkinsonism	Behavioral and Mood Changes	Time and Spatial Disorientation	Hemorrhage	Treatment	Evolution
Hurst et al., 1998 [26]	40/5	X						4 ET 1 ET and S	1 Death 4 Improved
Matsuda S., et al., 1999 [34]	3		X	X				ET	2 Improved 1 Not improved
Greenough et al., 1999 [49]	1	X						S	Improved
Abrahams., et al., 2002 [50]	1		X					ET	Improved
Magot A., et al., 2004 [27]	2	X	X					ET	Improved
Kwon., et al., 2005 [2]	27/1	X					X	ET	Improved
Chan H Y., et al., 2006 [51]	1	X		X	X	X	X	ET	Improved
Waragai M., et al., 2006 [43]	2		X					ET	Improved
Kajitani M et al., 2007 [52]	1		X	X		X		ET	Improved
Hasumi T et al., 2007 [23]	1	X				X		ET	Improved
Gonçalves MB et al., 2008 [25]	1	X				X		ET	Improved
Racine CA, et al., 2008 [28]	1	X						ET and S	Improved
Lv et al., 2008 [53]	3/1		X					ET	Improved
Nogueira RG., et al., 2009 [54]	1			X				ET and S	Improved
Wilson M, et al., 2010 [55]	3	X						ET	Improved
Netravathi M, et al., 2011 [42]	2	X		X				ET	Not improved
Geraldes et al., 2012 [38]	1	X		X				ET	Improved
Morparia N.., et al., 2012 [46]	1	X						ET	Improved
Hattori T., et al., 2013 [40]	1			X		X		ET	Improved
Iwasawa E., et al., 2013 [56]	1	X						ET	Improved
Jagtap SA., et al., 2014 [41]	1		X	X				NE	Death
Labeyrie MA., et al., 2014 [57]	45/8	X	X					ET	Improved
Chahbazian K., et al., 2014 [45]	1	X				X		ET	Improved
van Munster CE., et al., 2014 [24]	1	X			X			ET	Improved
Pasi M., et al., 2014 [58]	1	X						ET	Improved
Fujii H., et al., 2014 [32]	1		X	X				ET	Improved
Yoshihara., et al., 2014 [29]	1	X	X			X		ET	Improved
Imazeki R., et al., 2015 [59]	1		X					ET and S	Improved
Chen MA., et al., 2015 [47]	1	X			X			ET and S	Improved
Martínez Burbano, et al., 2016 [33]	1	X	X			X		NE	Death
Holekamp T F., et al., 2016 [48]	4	X	X		X			2 ET 2 ET and S	3 Improved 1 Death
Pu J., et al., 2017 [35]	1		X	X				ET	Improved
Lai J., et al., 2017 [60]	2	X		X	X			ET	Improved
Gopinath M., et al., 2017 [39]	1		X	X				ET	Improved
Enofe I., et al., 2017 [31]	1		X					ET	Improved
Zenteno M., et al., 2018 [30]	1	X				X		ET	Improved
Brito A., et al., 2019 [61]	389/6		X					ET	Improved
Current case report 2019	1				X	X		ET	

NE: natural evolution; ET: endovascular treatment; S: surgery.
